# No Time Dependence of Ciprofloxacin Pharmacokinetics in Critically Ill Adults: Comparison of Individual and Population Analyses

**DOI:** 10.3390/pharmaceutics13081156

**Published:** 2021-07-27

**Authors:** Martin Šíma, Danica Michaličková, Pavel Ryšánek, Petra Cihlářová, Martin Kuchař, Daniela Lžičařová, Jan Beroušek, Jan Miroslav Hartinger, Tomáš Vymazal, Ondřej Slanař

**Affiliations:** 1 Department of Pharmacology, First Faculty of Medicine, Charles University and General University Hospital in Prague, 128 00 Prague, Czech Republic; danica.michalickova@lf1.cuni.cz (D.M.); jan.hartinger@vfn.cz (J.M.H.); ondrej.slanar@lf1.cuni.cz (O.S.); 2 Forensic Laboratory of Biologically Active Substances, Department of Chemistry of Natural Compounds, University of Chemistry and Technology Prague, 166 28 Prague, Czech Republic; Petra.Cihlarova@vscht.cz (P.C.); Martin.Kuchar@vscht.cz (M.K.); 3 Department of Medical Microbiology, Second Faculty of Medicine, Charles University in Prague and Motol University Hospital, 150 06 Prague, Czech Republic; Daniela.lzicarova@lfmotol.cuni.cz; 4 Department of Anesthesiology and ICM, Second Faculty of Medicine, Charles University in Prague and Motol University Hospital, 150 06 Prague, Czech Republic; jan.berousek@fnmotol.cz (J.B.); tomas.vymazal@fnmotol.cz (T.V.)

**Keywords:** ciprofloxacin, pharmacokinetics, covariates, dosing, NONMEM, renal function

## Abstract

The aim of this prospective PK study was to evaluate the pharmacokinetics of ciprofloxacin dosed within the first 36 h (early phase) and after 3 days of treatment (delayed phase) using individual and population PK analysis. The secondary aim of the study was to evaluate possible dosing implications of the observed PK differences between early and delayed phases to achieve a PK/PD target for ciprofloxacin of AUC_24_/MIC ≥ 125. Blood concentrations of ciprofloxacin (1 and 4 h after dose and trough) were monitored in critically ill adults in the early and delayed phases of the treatment. Individual and population PK analyses were performed. Complete concentration-time profiles in the early phase, delayed phase, and both phases were obtained from 29, 15, and 14 patients, respectively. No systematic changes in ciprofloxacin PK parameters between the early and delayed phases were observed, although variability was higher at the early phase. Both individual and population analyses provided similar results. Simulations showed that after standard dosing, it is practically impossible to reach the recommended ciprofloxacin PK/PD target (AUC/MIC ≥ 125) for pathogens with MIC ≥ 0.5 mg/L. A dosing nomogram utilizing patients’ creatinine clearance and MIC values was constructed. Both individual and population analyses provided similar results. Therapeutic drug monitoring should be implemented to safeguard the optimal ciprofloxacin exposure.

## 1. Introduction

Antibiotic treatment is commonly used to eradicate bacterial infections in critically ill patients admitted to the intensive care unit. Ciprofloxacin is a wide-spectrum antibiotic that is commonly prescribed for various infections either in monotherapy or in combination with other antibiotics [1]. Its bactericidal action is distinguished by an activity primarily against Gram-negative aerobic bacteria, of which *Pseudomonas aeruginosa* and *Enterobacterales* are the most clinically important [2]. Specific pharmacokinetic/pharmacodynamic (PK/PD) target for ciprofloxacin is defined as the ratio of the 24 h area under the concentration-time curve (AUC_24_) over the minimum inhibitory concentration (MIC), where the MIC is determined as the lowest concentration of an antibiotic that prevents visible growth of bacteria in vitro [3]. Generally, attaining the PK/PD target of AUC_24_/MIC ≥ 125 should be predictive of sufficient anti-infective treatment. Nonetheless, AUC_24_/MIC ≥ 125 is usually not attained in critically ill patients treated with the standard recommended doses of ciprofloxacin (200–1500 mg/day), as shown in previous studies [4,5,6].

Both population and individual PK analyses are well-established and recognized types of PK explorations with distinct features that may lead to the preference for one procedure over another. In a population PK analysis performed using non-linear mixed-effects modeling (NLME), data of all patients included in the study are analyzed at the same time, providing estimates of population parameter values and inter- and intra-individual variability [7]. There are several advantages of the population PK analysis, such as the possibility to analyze sparse and unbalanced data, identification and quantification of predictive covariates, and ability to distinguish between inter- and intra-individual variability, or residual unexplained variability [7]. On the other hand, individual PK explorations are generally conducted to describe PK parameters and determine the most prominent factors with the highest potential to affect drug exposures. Although this individual approach leaves many possible sources of variability unexplored, the most important ones are generally better captured in a limited-sample-size population, provided a sufficient number of samples is obtained.

There is a high heterogeneity in the group of critically ill patients regarding the patients’ age, comorbidities, disease severity, pathogens and loci of infections [4,8]. Various pathophysiological factors in these patients usually lead to alteration of PK of the drugs. Altered PK of a drug may result in an inadequate exposure, causing insufficient bacterial eradication, an increased risk of antibiotic resistance, and surplus morbidity and mortality [9]. Therefore, detailed understanding of the PK of an individual antibiotic drug is necessary for its dosing optimization. Volume of distribution (Vd) can be increased due to the reduced protein binding, systemic inflammatory response syndrome (SIRS), and capillary leak [10]. Additionally, the elimination rate can be impaired or augmented, depending on the clinical condition of the patient [10]. SIRS can downregulate CYP enzymes, and this may lead to reduced clearance (CL) of drugs cleared by these enzymes [10]. On the other hand, CL of an antibiotic can be enhanced in sepsis, burn injury, or by concomitant use of inotropic agents [10].

Our hypothesis was that there might be a context-sensitive pharmacokinetic profile of ciprofloxacin as a result of complex instability of factors affecting ciprofloxacin PK in critically ill patients at the beginning of treatment, compared to likely more-stabilized conditions after 3 days of treatment.

The aim of this prospective study was therefore to evaluate the pharmacokinetics of ciprofloxacin dosed within first 36 h (early phase) and after 3 days of treatment (delayed phase). The secondary aim of the study was to evaluate possible dosing implications of the observed PK differences between the early and delayed phases to achieve a PK/PD target for ciprofloxacin of AUC_24_/MIC ≥ 125.

## 2. Materials and Methods

### 2.1. Study Design

This was a prospective, open-label (laboratory-blinded) pharmacokinetic study in adult patients treated with intravenous ciprofloxacin admitted to the Department of Anesthesiology and Intensive Care Medicine, Second Faculty of Medicine, Charles University in Prague, and Motol University Hospital between February 2019 and June 2020. The study was approved by the local Ethics Committee under No. EK 1492/18 on 2 January 2019 and was conducted in compliance with the Declaration of Helsinki. Written informed consent was obtained from all subjects before undertaking any study-related procedures. The study was registered in EudraCT under No. 2019-003732-24.

Ciprofloxacin was administered according to the standard clinical care in 30 min intravenous infusions of 400 or 600 mg every 8 or 12 h. The choice of dosing regimen was at the discretion of the clinician. Blood samples for the PK analysis were taken at 1, 4, and 7.5 or 11.5 h following completion of the infusion (the last sample was taken as a trough, depending on the dosing interval). This concentration-time profile was collected twice during the therapy—at the early phase (within 36 h after initiation of ciprofloxacin dosing) and at the delayed phase (72–96 h after initiation of ciprofloxacin dosing). Patients from whom at least one complete concentration-time profile was not collected were excluded from the study. Blood samples (5 mL) were collected via cannula into serum collecting tubes without clot activator and immediately placed in the cold. Samples were then centrifuged at 4500× *g* for 10 min at 4 °C, and serum aliquots were stored at −80 °C until analysis.

The following demographic, laboratory, and clinical features of patients were recorded as a potential covariates of ciprofloxacin pharmacokinetics: sex, age, body weight, height, smoking status, total bilirubin, serum creatinine, fluid balance, and co-medication with norepinephrine and furosemide. All clinical and laboratory parameters were determined separately on each sampling day. Hence, actual values were used for PK analysis both in the early and delayed phase.

Creatinine clearance (CL_CR_) was measured using serum creatinine (enzymatic assay) and 24 h urine output. CL_CR_ was calculated using the traditional equation CL_CR_ = U_CR_ × V/S_CR_, where U_CR_ is urine creatinine level (µmol/L), V is the urinary flow rate (mL/s), and S_CR_ is the serum level of creatinine (µmol/L) [11].

Glomerular filtration rate was also estimated using Chronic Kidney Disease Epidemiology Collaboration (CKD-EPI), Modification of Diet in Renal Disease (MDRD), Cockroft–Gault (C-G), and revised Lund–Malmö (L-M) formulas [12,13,14,15].

For each patient, body surface area (BSA) according to the DuBois formula and lean body mass (LBM) according to the Boer formula were calculated [16,17].

### 2.2. Bioanalytical Assay

Acetonitrile (ACN) and methanol (MeOH) (all LC-MS grade) were obtained from Honeywell (Charlotte, NC, USA). Ammonium acetate, formic acid, and ammonia solution 25% (all LC-MS grade) were supplied by Fluka (Buchs, Switzerland). Ultra-pure water was produced by a Smart2Pure system (Thermo Fisher Scientific, Waltham, MA, USA). Charcoal-stripped fetal bovine serum and ciprofloxacin (≥98%) were purchased from Sigma-Aldrich (Saint Louis, MO, USA), and internal standard ciprofloxacin-d8 (IS) from TRC (Totonto, ON, Canada). Standard stock solutions were prepared in water/acetic acid (60/40 *v*/*v*) at a concentration of 1 mg/mL and stored at −20 °C.

For serum samples, protein precipitation was used. Several procedures and reagents were tested; e.g., ACN, ACN/MeOH, and ACN/formic acid mixtures in several ratios. For the final procedure, 10 µL of IS solution (5 µg/mL) was added to 100 µL of serum and vortexed. Then, 200 µL of ice-cold mixture of ACN with 0.1% of formic acid was added and vortexed for 5 min. The procedure was repeated with another 200 uL of ACN/formic acid. The mixture was centrifuged at 13,000× *g* for 15 min, then 400 µL of the supernatant was evaporated and reconstituted in 500 µL of 10% methanol. All samples were prepared in triplicate.

The same procedure was used for the calibration curve; 100 µL of stripped bovine serum was spiked with ciprofloxacin standard solution to construct a calibration curve with the final range of 0.1–750 ng/mL.

For the UHPLC-MS/MS analysis, an Agilent 1290 Infinity UHPLC system coupled with an Agilent 6460 Triple quadrupole mass spectrometer (Agilent Technologies, Inc., Santa Clara, CA, USA) was used. Chromatographic separation was performed on a Kinetex EVO C18 column (2.1 × 50 mm; 1.7 µm) equipped with a guard column (both Phenomenex, Torrance, CA, USA). The mobile phases for gradient elution were ammonium acetate in water (0.5 mM) with ammonia solution, pH adjusted to 10.5 (A) and methanol (B). The flow rate was 0.45 mL/min and column temperature 30 °C. Gradient elution was carried out as follows: 0 min, 90:10 (A:B); 2 min, 60:40; 3.5 min, 0:100; 4.5 min, 0:100; 4.7 min, 90:10; 6.5 min, 90:10.

The MS/MS apparatus was operated in positive mode. The applied conditions of the electrospray ion source were: drying gas temperature 300 °C; drying gas flow 13 L/min; sheath gas temperature 350 °C; sheath gas flow 11 L/min; nebulizer pressure 40 psi; nozzle voltage 0 V; capillary voltage 2500 V. Multiple reaction monitoring (MRM) mode was used for the detection. Two transitions of *m*/*z* were used: 332.14 → 314.1 and 231 for ciprofloxacin and 340.19 → 322.2 and 235 for ciprofloxacin-d8.

An Agilent Mass Hunter (Agilent Technologies, Inc., Santa Clara, CA, USA) was used for data acquisition and quantification of samples.

### 2.3. Primary PK Analysis

Individual ciprofloxacin pharmacokinetic parameters—Vd, CL, and elimination half-life (t_1/2_)—were calculated in a one-compartmental pharmacokinetic model with first-order elimination kinetics based on individual demographic and clinical data and observed ciprofloxacin serum levels using MWPharm^++^ software version 1.8.2 (MediWare, Prague, Czech Republic). The ciprofloxacin PK data derived from Drusano et al. was used for an a priori estimation of the concentration-time profile in each patient [18]. These estimated PK profile curves were a posteriori individualized to maximize fitting with observed concentration points in each patient. The fitting was performed using Bayesian method separately for both the early and delayed phase concentrations set. The goodness of fit was expressed using weighted sum of squares and root mean square values.

Subsequently, a Mann–Whitney U-test and linear regression model were used to evaluate the relationships between ciprofloxacin individual PK parameters with categorical and continuous variables, respectively. PK parameters and measured CL_CR_ obtained from early and delayed concentration-time profiles were compared using a Mann–Whitney U-test (for this analysis, only patients with both complete phase profiles were included). GraphPad Prism software version 8.2.1 (GraphPad Inc., La Jolla, CA, USA) was used for all comparisons, and *p*-levels < 0.05 were considered as statistically significant.

### 2.4. Population PK Analysis

Population PK analysis was performed using NONMEM version 7.3.0 (ICON Development Solutions, Ellicott City, MD, USA) and PsN v3.4.2 [19,20] both running under Pirana 2.9.0 [21]. Modeling was carried out using the first-order conditional estimation method with interaction (FOCE-I). R 3.3.2 was used for the visualization of the data and model diagnostics.

Model development was performed in three steps:

(1) Development of structural and statistical model

For the structural model, one- and two-compartment models were tested to describe the distribution of ciprofloxacin. First-order clearance of ciprofloxacin was assumed. Log-normally distributed inter-individual variability terms with estimated variance were tested on each PK parameter. As change in the clinical status of the patients between the early phase and delayed phase of ciprofloxacin treatment was expected, inter-occasional variability was also tested. Proportional, additive, and combination error models were tested for the residual error model.

(2) Covariate analysis

The following variables were tested as covariates (characteristics predictive of inter-individual variability):

- Body weight, height, LBM, BSA, serum level of bilirubin, CL_CR_, age, daily fluid balance, and doses of concomitantly used drugs (noradrenalin and furosemide) were tested as continuous covariates;
- Smoking status (smoker/non-smoker), concomitant therapy with continuous veno-venous hemodialysis—CVVHD (on CVVHD/off CVVHD) and sex were tested as categorical covariates.

A stepwise covariate modeling procedure was performed. Continuous covariates were tested in linear and power functions. Categorical covariates were tested by estimating the parameter value for one category as a fraction of the parameter value for the other category. For model selection, a decrease in objective function value (OFV) of more than 3.84 points between nested models (*p* < 0.01) was considered statistically significant, assuming a χ^2^ distribution. Additional criteria for model selection were relative standard error (RSE) of the estimates of structural model parameters <30%, physiological plausibility of the obtained parameter values, and absence of bias in goodness-of-fit (GOF) plots.

(3) Validation of the final model

To evaluate the stability of the model, a bootstrap analysis was performed. In this procedure, 1000 replicates of the original data were generated, and the parameter estimates for each of the 1000 samples were re-estimated by NONMEM in the final model. The median and 95% confidence intervals (CI) obtained for each parameter estimated for bootstrap samples were compared with the estimates in the final model. The predictive properties of the structural and statistical model were validated using normalized prediction distribution errors (NPDEs). For this, the dataset was simulated 1000 times, after which the observed concentrations were compared to the range of simulated values using the NPDE package developed for R [22]. Additionally, a visual predictive check (VPC) was performed to evaluate the predictive accuracy of the final model [23]. For this, 1000 replicates of the original dataset were simulated using the final model parameter estimates, and the simulated distribution was compared with that from the observed data. The 95% CIs for the 10th, 50th, and 90th percentiles of the simulations were calculated from all replicates and presented graphically.

#### Monte Carlo Simulations

Monte Carlo simulations (*n* = 1000) were performed to assess the probability of target attainment (PTA) of the PK/PD target for ciprofloxacin (AUC/MIC ratio > 125) for various MICs (0.0625 to 1 mg/L). Standard dosing regimens consisting of 400 mg b.i.d. and t.i.d were simulated for different levels of CL_CR_ (0.5, 1, 1.5 mL/s). Dosing regimen was regarded to be successful if the PTA was >100%.

## 3. Results

### 3.1. Study Population

There were 35 patients enrolled in the study. Five patients were excluded due to deviations in sampling times or missing samples. Complete concentration-time profile in the early phase, delayed phase, and both phases was obtained from 29, 15, and 14 patients, respectively. The main reasons for not completing both study phases were discontinuation of ciprofloxacin therapy, patient transfer from the intensive care unit, or significant deviations from sampling schedule. Demographic, laboratory, and clinical characteristics of patients are summarized in Table 1. The most frequent indications for ciprofloxacin use were acute exacerbations of chronic obstructive pulmonary disease, lower respiratory tract infections, and soft tissue infections. Among patients included in the PK analysis (*n* = 30), only one subject received CVVHD support, and none were treated with extracorporeal membrane oxygenation. Ciprofloxacin dose ranged from 800 mg/day (400 mg every 12 h) to 1200 mg/day (400 mg every 8 h or 600 mg every 12 h). In total, 132 ciprofloxacin serum concentrations were included in the analysis.

### 3.2. Primary PK Analysis

Individual pharmacokinetic parameters of ciprofloxacin are summarized in Table 2. Median (interquartile range) weighted sum of squares and root mean square values were 7.43 (2.60–10.98) and 0.96 (0.91–0.98), respectively. We observed medium to high inter-individual variability of PK parameters normalized per kg of body weight demonstrated by coefficients of variation of 46%, 74%, and 37% for Vd, CL, and t_1/2_, respectively (early-phase group). On the contrary, there were no significant differences either in ciprofloxacin PK parameters or in measured CL_CR_ between the early and delayed phases (*p*-values of 0.2798, 0.6673, 0.7088, and 0.5189 for body-weight-normalized Vd, CL, t_1/2_, and CL_CR_, respectively).

Both ciprofloxacin Vd and CL were significantly and positively related with CL_CR_ (*p* = 0.0009 and *p* < 0.0001, respectively) and negatively related with age (*p* < 0.0001 for both Vd and CL). Since CL_CR_ significantly decreased with increasing age (*p* < 0.0001), we assumed that the real independent variable was only CL_CR_. Measured CL_CR_ was the most predictive of the ciprofloxacin CL (r^2^ = 0.6275), but estimated glomerular filtration rates according to various formulas were also significantly related to ciprofloxacin CL (r^2^ of 0.4342, 0.4301, 0.4088, and 0.3701 according to CKD-EPI, L-M, MDRD, and C–G equation, respectively). The weakest predictive performance for estimating of ciprofloxacin CL was observed in serum creatinine level (r^2^ = 0.1717). These relations are presented in Figure S1. Ciprofloxacin CL was also negatively related to the daily dose of norepinephrine (*p* = 0.0249), but analogically, since the dose of norepinephrine was associated with CL_CR_ (*p* = 0.0060), we assumed that the only dependent variable and the real covariate was CL_CR_. Both ciprofloxacin Vd and CL were also positively related with height (*p* = 0.0263 and *p* = 0.0240, respectively). PK of ciprofloxacin was neither related to body weight nor to any derived body-size descriptors such as BSA and LBM. We also observed no relationships with smoking status, serum level of bilirubin, fluid balance, or daily dose of furosemide. There were no differences in the ciprofloxacin PK between the sexes.

**Figure 1:**
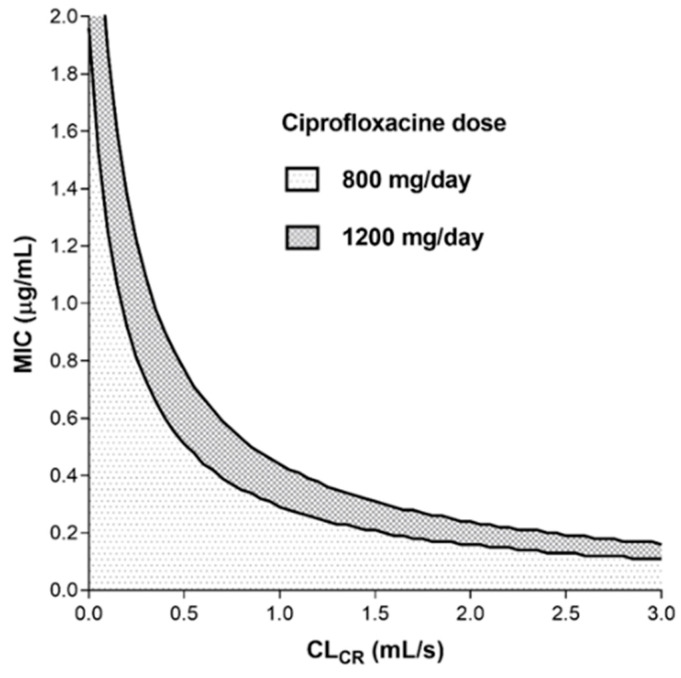
Nomogram representing the relation between patient creatinine clearance (CL_CR_), minimum inhibitory concentration for the pathogen (MIC), and ciprofloxacin PK/PD target attainment (AUC_24_/MIC ratio of 125) at the usual ciprofloxacin dosage (800 or 1200 mg/day). The white area of the graph corresponds to renal clearance and MIC values, for which the standard dosing could not achieve the PK/PD target.

Based on the strongest observed relationship (ciprofloxacin CL = 18.54 × CL_CR_ + 3.261; r^2^ = 0.6275) and the well-known equation (AUC = dose/CL), we constructed a nomogram (Figure 1) representing the relation between patient CL_CR_, MIC for pathogen, and ciprofloxacin PK/PD target attainment (AUC_24_/MIC ratio of 125) at the usual ciprofloxacin dosage (800 or 1200 mg/day).

### 3.3. Population PK Analysis

Observed ciprofloxacin plasma concentrations were best described by a one-compartment model with log-normally distributed intra-individual variability on CL and Vd. A combination residual error model provided the best description of residual variability. Inter-occasion variability tested as a third level of random effects was not found to be significant.

Inclusion of CL_CR_ in a linear relationship as a covariate on CL resulted in a statistically significant improvement of the model fit (*p* < 0.001). Inclusion of this covariate in the model led to a decrease in inter-individual variability of CL from 71.6% (basic model without covariates) to 44.9% (final model). None of the other covariates were statistically significant.

The final parameter estimates are presented in Table 3. In the final model, for a typical individual with CL_CR_ of 1.25 mL/s, CL and Vd were 21.5 L/h and 143 L (34%), respectively.

**Figure 2:**
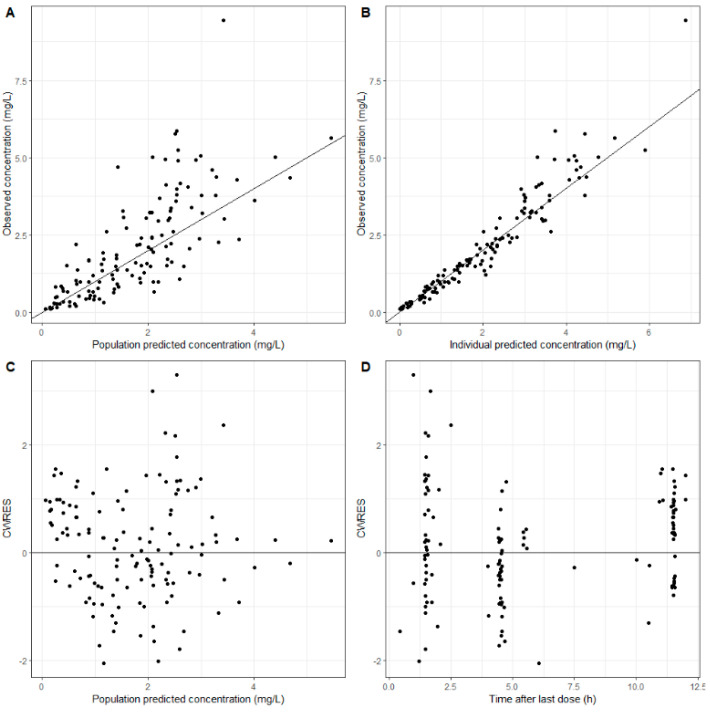
Goodness-of-fit plots for the final model for ciprofloxacin concentrations in critically ill patients. (**A**) Population-predicted ciprofloxacin concentrations vs. observed ciprofloxacin concentrations. (**B**) Individual-predicted ciprofloxacin concentrations vs. observed ciprofloxacin concentrations. (**C**) Conditional weighted residuals (CWRES) vs. population-predicted ciprofloxacin concentrations. (**D**) Conditional weighted residuals (CWRES) vs. time after last dose.

The precision of the estimated structural parameter values was acceptable, with RSE values below 30%. The basic GOF plots in Figure 2 indicate that the final model could describe the data accurately, as the predicted population and predicted individual concentrations were described without bias. All median parameter values in the bootstrap procedure were within 10% of the values obtained in the final model fit, indicating that the model was robust.

**Figure 3:**
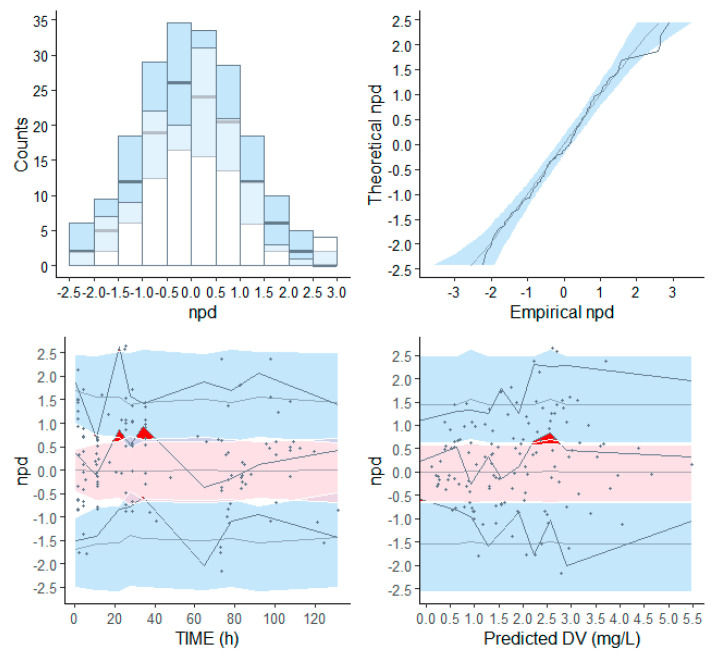
Visual output of the normalized prediction distribution error (NPDE) analysis. Shown at the top are the QQ plot and histogram of the NPDEs in the overall dataset. The red dotted line and blue shaded areas show the expected trends and 95% confidence intervals of these trends, while the dark blue lines and bars show the observed NPDE distributions. At the bottom, the individual NPDE values for each observation are plotted versus time and versus the predicted concentrations with the symbols. The solid lines in the bottom graphs indicate the mean (red) and the 95% percentiles (blue) of the NPDEs, and the shaded areas are the simulated 95% confidence intervals of the NPDE median (red) and 95% percentiles (blue), while the dotted red and blue lines show the expected values for the median and 95% percentiles.

The distribution of the NPDEs obtained with the model for the dataset had a mean of 0.039 (SE = 0.087) and variance of 1 (SE = 0.12). Neither of these values were significantly different from the expected values of 0 (*p* = 1) and 1 (*p* = 1), respectively (Figure 3). This indicates that predictions regarding the structural model and the variability in the data were accurate. Adequate performance of the final model was confirmed with the VPC analysis (Figure S2). However, the VPC showed that the median of the observed concentrations did not fall into the CI of median prediction (red shaded area); therefore, caution should be taken when using this model for extrapolation to early time points after dosing.

#### Monte Carlo Simulations

**Figure 4:**
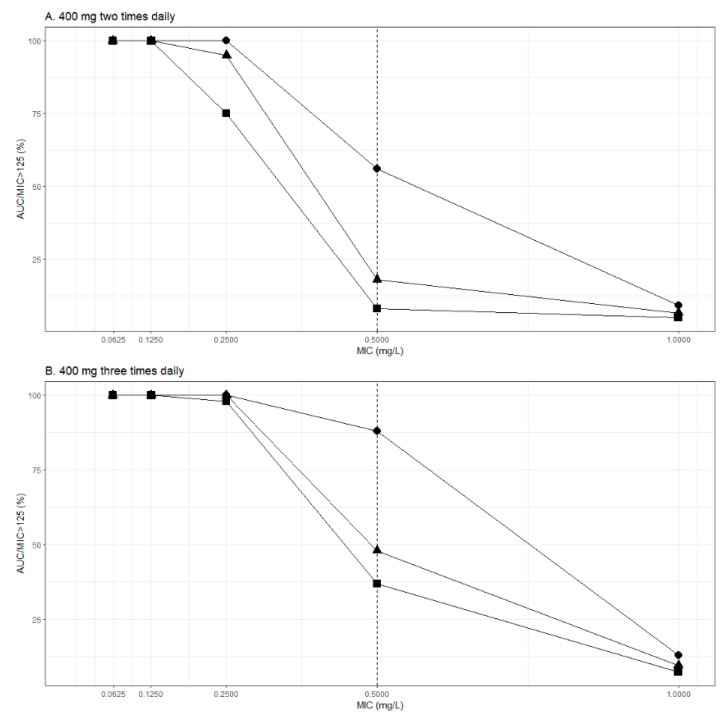
Monte Carlo simulations for ciprofloxacin PTA (AUC/MIC > 125) at different creatinine clearance (CL_CR_) values. (**A**) 400 mg two times daily and (**B**) 400 mg three times daily.

Figure 4 shows the PTA values for different dosing regimens (400 mg b.i.d. and t.i.d.) of ciprofloxacin for various MIC values (0.0625–1 mg/L) for patients with different values of CL_CR_ (0.5, 1, 1.5 mL/s). Dosing regimen including 400 mg b.i.d. (Figure 4A) was sufficient for MICs ≤ 0.125 mg/L, but was insufficient for MICs ≥ 0.25 mg/L, except for impaired renal function (CL_CR_ = 0.5 mL/s). Dosing regimen consisting of ciprofloxacin t.i.d. (Figure 4B) enabled PTA > 100% for MIC ≤ 0.25 mg/L. No approved dosing regimen achieved sufficient PTA for MICs ≥ 0.5 mg/L.

## 4. Discussion

Variability in the clinical status of the patients between the early phase and delayed phase of ciprofloxacin treatment was expected, therefore inter-occasion variability was tested. However, no significant differences of ciprofloxacin PK parameters between the early and delayed phases of treatment was observed. Similarly, inter-occasion variability tested as a third level of random effects in the population PK analysis, and it was not significant. Since shifts in body fluid have been implicated as a major cause of alteration in distribution [24], it can be assumed that especially Vd of hydrophilic drugs may be altered. Ciprofloxacin is lipophilic, and as such it is likely to be less susceptible to changes in distribution during critical illness; however, our study could not precisely describe the distribution phase. Substantial changes in functional of eliminating organs resulting in significant variability of a drug CL are also common in critically ill patients [24]. In our study population, we consistently observed higher spread (both increase and decrease) of renal-function status in the early phase in comparison with the delayed phase. However, median CL_CR_ values were without statistically significant difference (*p* = 0.5189) in both phases, and thus no significant difference in ciprofloxacin CL between the early and delayed phase was also noted.

In a recent position paper, individualized antibiotic dosing was unambiguously recommended for aminoglycosides, glycopeptides, beta-lactams, and linezolid, while for ciprofloxacin (fluoroquinolones), the therapeutic drug monitoring was neither recommended nor discouraged [25]. The nomogram based on individual PK analysis (Figure 1) shows that at standard dosage (800–1200 mg/day), it was practically impossible to reach the recommended ciprofloxacin PK/PD target (AUC/MIC ≥ 125) when the MIC ≥ 0.5 mg/L (except for patients with moderate to severe renal impairment). This observation was consistent with ciprofloxacin-resistance breakpoint value of 0.5 mg/L stated by the European Committee on Antimicrobial Susceptibility Testing (EUCAST). On the other hand, the ciprofloxacin PK/PD target should be reached at standard dosing when the MIC is less than 0.25 mg/L (except in patients with augmented renal clearance), which fully corresponds with the EUCAST-sensitivity breakpoint value for ciprofloxacin. However, it is evident from the nomogram that in patients with augmented renal clearance, even a ciprofloxacin dose of 1200 mg/day may not be effective, and on the contrary, commonly used doses may lead to overexposure in patients with renal impairment.

Monte Carlo simulations were performed to assess PTA of the ciprofloxacin PK/PD target (AUC/MIC ≥ 125). Dosing regimen including 400 mg b.i.d. was sufficient for MICs ≤ 0.125 mg/L but was insufficient for MICs ≥ 0.25 mg/L, except for patients with impaired renal function (CL_CR_ = 0.5 mL/s). Additionally, a dosing regimen consisting of ciprofloxacin 400 mg t.i.d. enabled PTA > 100% for MICs ≤ 0.25 mg/L. Finally, no dosing regimen achieved sufficient PTA for MICs ≥ 0.5 mg/L. Similar results were obtained in the previous studies conducted in critically ill patients [4,26]. Roberts et al. showed that for a directed therapy against *P. aeruginosa* (MIC value of 0.5 mg/L), a dose of 600 mg t.i.d. would be needed to achieve an adequate PTA in patients with septic shock [26]. Although simulations show daily doses > 1200 mg would be required for optimal exposure of ciprofloxacin for MICs ≥ 0.5 mg/L, it is important to note that these dosing regimens have never been prospectively and externally validated. Higher doses increase risk of unwanted effects, which may go undiscovered in critically ill patients who are predisposed to similar adverse effects due to co-medication or their underlying illness. Relatively high incidence of both augmented renal clearance and impaired renal function status among critically ill patients support the clinically reasonable approach to implement therapeutic drug monitoring of fluoroquinolones to safeguard efficacious and safe antimicrobial treatment in this vulnerable patient population.

We can distinguish between the individual and population approaches to perform PK analyses. Advantages and disadvantages of these approaches or their suitability for specific situation are often discussed, but there is a lack of direct comparison of these methods on the same dataset [27]. Population approach offers significant advantages with respect to comprehensive PK exploration and drug-dosing posology in the population, while the individual approach is superior in terms of the routine clinical care of an individual patient. We analyzed data from our study using both individual and population PK approaches. The median values of ciprofloxacin PK parameters from individual analysis were almost the same as Vd and CL values for a typical individual in the final population model. CL_CR_ also proved to be the main independent covariate of ciprofloxacin CL in both analyses. In individual analysis CL_CR_ was also associated with ciprofloxacin Vd. This observation can be explained by fluid retention in patients with impaired renal function resulting in a relative decrease in distribution space for lipophilic agents such as ciprofloxacin. Relation of PK parameters with body size is common, but body weight is usually the strongest covariate, especially in lipophilic drugs [28]. Since we observed only a weak relationship with height in the individual PK analysis and the other body size descriptors were without significance, it might be considered as a chance finding.

We also compared glomerular filtration rate (measured CL_CR_) with multiple estimation equations (CKD-EPI, L–M, C–G, and MDRD) and simple serum creatinine level as determinants of ciprofloxacin CL. As expected, the best predictive performance was shown for measured CL_CR_ (r^2^ = 0.6275). The CKD-EPI or L–M showed numerically better performance in comparison with MDRD, C–G, or simple serum creatinine level, which was in accordance with previous observations in other renally excreted antibiotic agents [29,30]. The MDRD formula is applicable primarily in chronic kidney disease subjects. The C–G equation includes body weight, which can lead to a distortion in under- or overweight patients. This may be the reason for the worse performance of glomerular filtration rate calculated by the MDRD or C–G formulas when used in a heterogeneous population. However, estimation of glomerular filtration rate by means of any equation may be reliable only in patients with a stable renal function. Therefore, measurement of CL_CR_ is considered the “gold standard” in the routine care of patients with unstable renal functions, and estimation methods will likely not be used in the patient population included in our study.

For the individual PK analysis, we used a two-stage method, in the first stage of which the values of the PK parameters in each patient were calculated. Although we used mean (SD) from Drusano et al. as a Bayesian prior [18], the PK parameters were adapted as random variables via a large number of iterations to achieve maximum fitting of the simulated PK profile with the true observed concentration points in each individual. The fitting was very tight, as evidenced by the goodness-of-fit values. In the second stage, the individual PK parameters were associated with patient characteristics using regression models (continuous variables) or column statistics (categorical variables). The values of PK parameters obtained from this analysis corresponded well with data from other ciprofloxacin individual PK studies [31,32,33,34].

Although a direct comparison of findings between population PK studies using a NLME approach is difficult due to differences in parameterization and covariate relationships, it is possible to make comparisons between parameter values for typical individuals. So far, two studies using NLME modeling to describe PK ciprofloxacin in critically ill patients have been published [4,26]. One of these studies (Roberts et al.) was conducted in patients with sepsis [26]. To allow comparison of PK parameters, we calculated parameter values for the typical individual from our study with CL_CR_ of 1.25 mL/s using the provided equations in the corresponding publications. The estimated values of CL in this study for a typical individual (21.5 L/h) were similar to the values reported in the previous studies (25 L/h according to Abdulla et al. [4], and 15 L/h according to Roberts et al. [26]). Moreover, Roberts et al. [26] found CL_CR_ to be a significant covariate for CL, and this relationship was described as a power function. In our study, this relationship was found to be linear. On the other hand, Abdulla et al. [4] found no significant covariates for CL. Regarding Vd, we found no predictive covariates, similarly to Abdulla et al. [4]. Conversely, Roberts et al. [26] reported body weight to be a predictive covariate for central Vd.

The studies by Abdulla et al. [4] and Roberts et al. [26] found the two-compartment model to provide the best description of ciprofloxacin disposition, whereas we selected a one-compartment model. These differences can be explained by a relatively sparse blood sampling at early time points after onset of treatment with ciprofloxacin in the current study, therefore caution should be taken when using this model for extrapolation to early time points after dosing. This was also confirmed by the VPC, as the median of the observed concentrations did not fall into the CI of the median prediction in the early time points after the first dose of ciprofloxacin.

## 5. Conclusions

In conclusion, we described PK of ciprofloxacin using both individual and population approaches and obtained similar results. We observed no changes in ciprofloxacin PK parameters between the early and delayed phases of treatment. CL_CR_ (as a marker of functional renal status) was identified as a significant covariate of ciprofloxacin PK. Standard dosing regimens might be insufficient for achieving the optimal therapeutic target, therefore therapeutic drug monitoring should be implemented to increase the probability of optimal ciprofloxacin exposure and to avoid unnecessary high concentrations.

## Figures and Tables

**Table 1:** Demographic, laboratory, and clinical characteristics of patients (*n* = 30).

Characteristics	Early Phase (*n* = 29)	Delayed Phase (*n* = 15)
Sex (M/F)	21/8	11/4
Age (years)	58 (35–85)	57 (35–85)
Body weight (kg)	90 (56–140)	90 (58–130)
Height (cm)	175 (150–196)	175 (160–190)
BSA (m^2^)	2.09 (1.60–2.65)	2.09 (1.60–2.45)
LBM (kg)	63 (44–81)	63 (44–76)
Smoking status (Y/N/NA)	15/6/8	9/3/3
CL_CR_ (mL/s)	1.16 (0.12–3.32)	1.36 (0.66–2.49)
CKD-EPI (mL/s)	1.60 (0.16–2.98)	1.24 (0.76–2.37)
MDRD (mL/s)	1.70 (0.18–4.26)	1.99 (0.94–4.95)
C–G (mL/s)	1.77 (0.25–4.96)	2.10 (0.72–5.31)
L–M (mL/s)	1.45 (0.20–2.81)	1.76 (0.82–2.95)
Total bilirubin (µmol/L)	11.3 (3.5–90.3)	11.0 (4.4–46.4)
Fluid balance (mL/day)	−490 (−4000–4300)	−500 (−3502–1900)
Norepinephrine (mg/day)	17 (0–61)	5 (0–60)
Furosemide (mg/day)	5 (0–320)	19 (0–148)

Data are presented as median (range). BSA—body surface area; LBM—lean body mass; CL_CR_—measured creatinine clearance; M—men; W—women; Y—yes; N—no; NA—not available; CKD-EPI—Chronic Kidney Disease Epidemiology Collaboration; MDRD—Modification of Diet in Renal Disease; C–G—Cockroft–Gault; and L–M—revised Lund–Malmö equations for estimation of glomerular filtration rate.

**Table 2:** Individual pharmacokinetic parameters of ciprofloxacin.

PK Parameter	Early Phase (*n* = 29)	Delayed Phase (*n* = 15)
Vd (L)	136.9 (76.6–322.6)	158.3 (98.0–386.6)
Vd (L/kg)	1.73 (0.76–3.83)	1.98 (0.75–4.55)
CL (L/h)	18.59 (8.13–74.94)	20.57 (6.20–74.63)
CL (L/h/kg)	0.209 (0.068–0.906)	0.291 (0.107–0.878)
t_1/2_ (h)	5.6 (2.3–11.7)	4.7 (2.8–11.6)

Data are presented as median (range). Vd—volume of distribution; CL—clearance; t_1/2_—elimination half-life.

**Table 3:** Parameter estimates of the final model for ciprofloxacin in critically ill patients.

Parameter (Units)	Final Model (RSE %)	Bootstrap Method (95% CI)
Fixed effects		
CL (L/h) = CL_p_ + θCL_CR_ × (CL_CR_/1.25)		
CL_p_ (L/h)	5.4 (28%)	5.33 (2.21–8.50)
θCL_CR_	16.1 (16%)	16 (11.3–21.8)
Vd (L) = Vd_p_		
Vd_p_ (L)	143 (9%)	142 (112–168)
Inter-individual variability		
CL (%)	44.9% (3%)	43.6% (34.9–52.4)
Vd (%)	34.8% (17%)	43.6% (34.9–52.4)
Residual variability		
Additive (%)	0.981 (34%)	1.05 (0.6–2.1)
Proportional (%)	4.78 (26%)	4.5 (2.3–7.5)

RSE—relative standard error of the estimate; CL—clearance; CL_p_—population clearance value; CL_CR_—creatinine clearance; θCL_CR_—increase in CL per L/s CL_CR_; Vd—volume of distribution; Vd_p_—population volume of distribution value.

## Data Availability

The data that support the findings of this study are available from the corresponding author upon reasonable request.

## Supplementary Materials

The following are available online at https://www.mdpi.com/article/10.3390/pharmaceutics13081156/s1, Figure S1: Relationships of ciprofloxacin clearance (CL_CIP_) with creatinine serum concentrations, with measured creatinine clearance (CL_CR_), and with glomerular filtration rate estimations according to Chronic Kidney Disease Epidemiology Collaboration (CKD-EPI), Modification of Diet in Renal Disease (MDRD), Cockroft-Gault (C-G), and revised Lund-Malmö (L-M) equations, Figure S2: Visual predictive check of the final model. Black dots—observations, black dashed lines - 80%-interval and median of the observations, red shaded area—95%-confidence interval (CI) of the median prediction, blue shaded area—95%-CI of the 10 and 90th prediction interval.
