# Supplementary material for: No Time Dependence of Ciprofloxacin Pharmacokinetics in Critically Ill Adults: Comparison of Individual and Population Analyses
Source: Pharmaceutics. 2021 Jul 27;13(8):1156. doi: 10.3390/pharmaceutics13081156 (PMC8402031; doi:10.3390/pharmaceutics13081156)
Supplement: Supplementary file 1 [file pharmaceutics-13-01156-s001.zip › pharmaceutics-1274054-supplementary.pdf]

pharmaceutics

MDPI

Supplementary Materials: No Time Dependence of Ciprofloxacin Pharmacokinetics in Critically Ill Adults: Comparison of Individual and Population Analyses

Martin Šíma, Danica Michaličková, Pavel Ryšánek, Petra Cihlářová, Martin Kuchař, Daniela Lžičařová, Jan Beroušek, Jan Miroslav Hartinger, Tomáš Vymazal and Ondřej Slanař

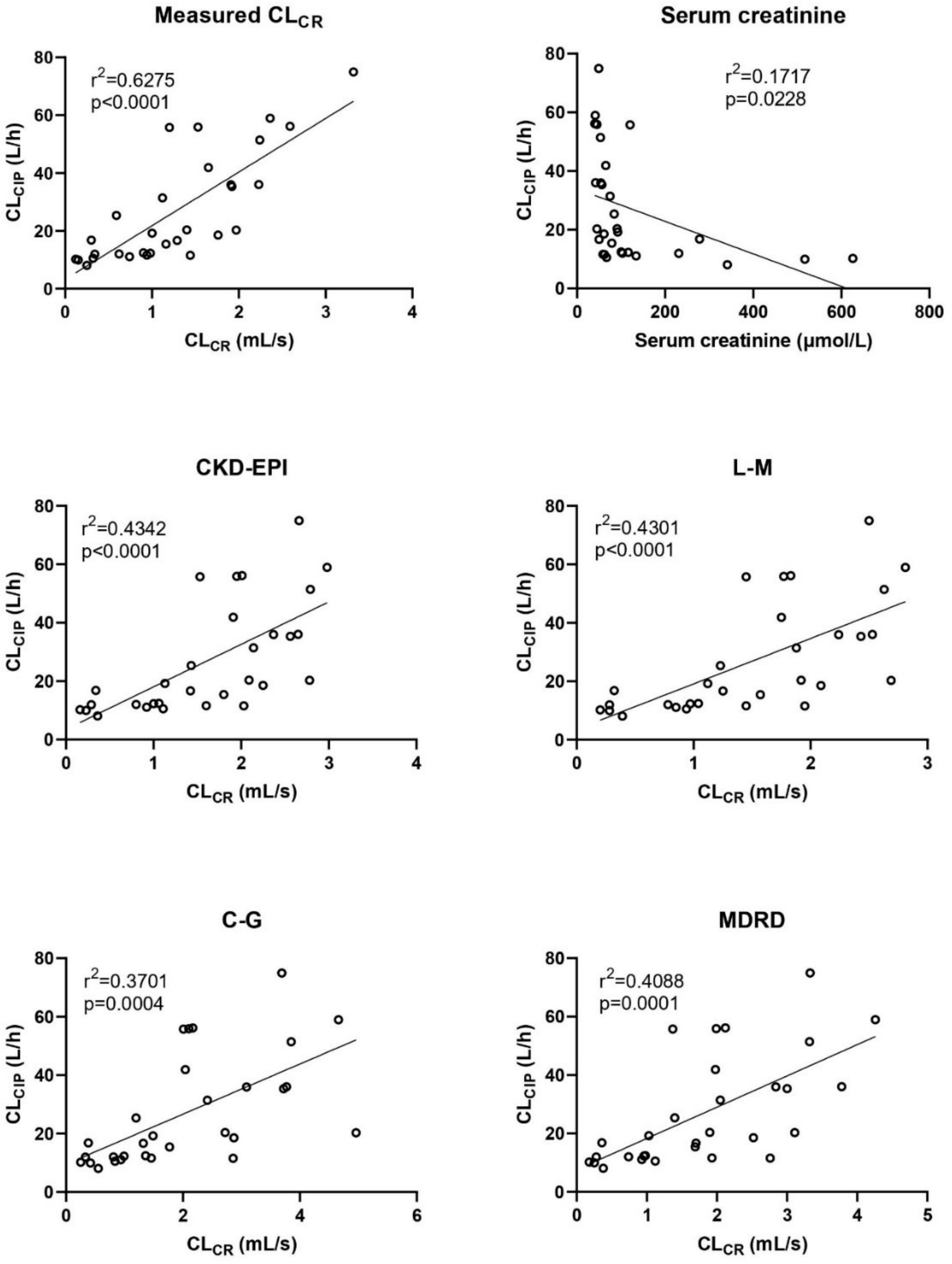

Figure S1. Relationships of ciprofloxacin clearance (CLCIP) with creatinine serum concentrations, with measured creatinine clearance (CLCR), and with glomerular filtration rate estimations according to Chronic Kidney Disease Epidemiology Collaboration (CKD-EPI), Modification of Diet in Renal Disease (MDRD), Cockroft-Gault (C-G), and revised Lund-Malmö (L-M) equations.

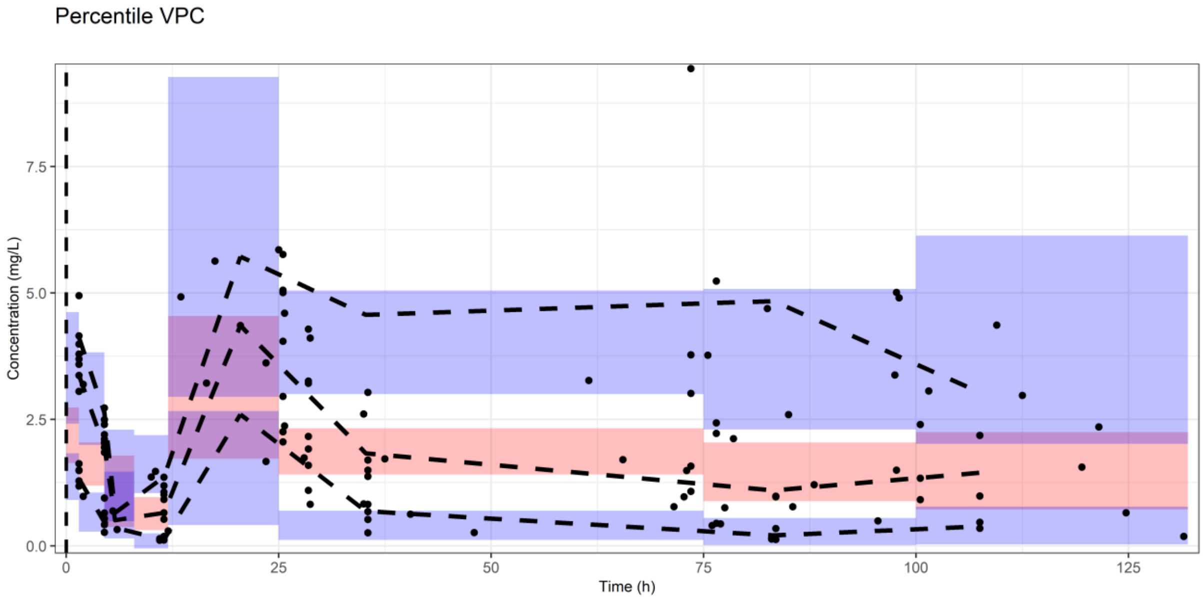

Figure S2. Visual predictive check of the final model. Black dots - observations, black dashed lines - 80%-interval and median of the observations, red shaded area - 95%-confidence interval (CI) of the median prediction, blue shaded area - 95%-CI of the 10 and 90th prediction interval.
